# Vulnerability of Myrmecochory to Anthropogenic Disturbances and Climate Change: An Ecological Synthesis

**DOI:** 10.3390/insects17070677

**Published:** 2026-06-29

**Authors:** Seongwon Yun, Sle-gee Lee, Dong-Pyeo Lyu, Kyeong-Sik Cheon, Yoon Young Lee, Tae Kyung Yoon

**Affiliations:** 1 Department of Forest Science, Sangji University, Wonju 26339, Republic of Korea; honeybee0426@naver.com (S.Y.); myrmicinae@sangji.ac.kr (D.-P.L.); 2 Korea Environment Institute, Sejong 30147, Republic of Korea; cristallize@naver.com; 3 Department of Biological Science, Sangji University, Wonju 26339, Republic of Korea; cheonks@sangji.ac.kr; 4 Hwanghaksan Arboretum, Yeoju 12653, Republic of Korea; ylee14@korea.kr; 5 Department of Forestry, Environment, and Systems, Kookmin University, Seoul 02707, Republic of Korea

**Keywords:** ant-plant interaction, elaiosome, mutualism, seed dispersal, zoochory

## Abstract

Myrmecochory is a form of seed dispersal mediated by ants. Although this mechanism of dispersal has received less research attention than other dispersal processes, the wide distribution and high biomass of ants mean that it can strongly influence plant dispersal patterns. In particular, the underlying mechanisms and key agents of myrmecochory remain poorly understood in the context of anthropogenic perturbations; furthermore, such research is especially scarce in East Asia. This review aims to elucidate the ecological mechanisms underlying myrmecochory, to explore how this interaction may be affected by urbanization and climate change, and to determine its potential ecological role in disturbed ecosystems. We first review past research on the three major hypotheses proposed for the emergence of ant-mediated seed dispersal—directed dispersal, distance dispersal, and predator avoidance. We then compile taxonomic information on myrmecochorous plants and ants from global databases and regional literature, expanding the checklist of Korean myrmecochorous plants to 130 species and reclassifying them as endangered, rare, or endemic. Our synthesis suggests that invasive ants could threaten myrmecochory by displacing native myrmecochorous ants, increasing seed predation, and facilitating the dispersal of invasive plants. Moreover, the urban heat island effect and habitat fragmentation could disturb the dispersal, germination, and growth of myrmecochorous plants, threats that may be further intensified by climate-driven phenological mismatches. Consequently, in temperate East Asian countries experiencing anthropogenically generated environmental changes, myrmecochory emerges as a pivotal ecological process that underscores ecosystem vulnerability and resilience. Ultimately, incorporating these plant–ant interactions into biodiversity monitoring is essential for predicting ecosystem shifts and designing robust, proactive conservation strategies in changing environments.

## Simple Summary

Seed dispersal through ant-plant interactions is a fascinating ecological process, yet it has received less attention than other plant-insect relationships. This review synthesizes the ‘who, where, what, how, and why’ of seed dispersal by ants. While this mechanism has been predominantly studied in the Southern Hemisphere, there is a critical need for more research in temperate East Asia, a region currently facing rapid human-driven environmental changes such as urbanization, climate change, and biological invasions. Ultimately, ant-mediated seed dispersal may exhibit both vulnerability and resilience to human perturbations, depending on specific ecological contexts.:

## 1. Introduction

Plant dispersal has evolved in response to various environmental factors and biotic agents, as seeds generally lack autonomous mobility. In contrast to autochorous mechanisms—such as gravity-driven dispersal (barochory) and explosive dehiscence (ballochory)—allochorous strategies have commonly evolved with the aid of external factors, including wind (anemochory), water (hydrochory), and animal-mediated vectors (zoochory) [1,2]. Among these, seed dispersal by ants, termed myrmecochory, may offer unique ecological advantages that contribute to seed dispersal success—advantages that are often not provided by other forms of zoochory such as the attachment of seeds to an animal’s exterior (epizoochory) or seed ingestion followed by defecation (endozoochory) [3]. However, even though myrmecochory is recognized as a significant ant–plant interaction and can play an important role in plant spatial distributions and dispersal patterns, it has received less research attention than other seed dispersal strategies [4]. This is a particular concern because it is important to understand myrmecochory in order to assess the ecological impacts of invasive myrmecochorous plants and ants.

Historically, the ‘who, where, what, how, and why’ of seed dispersal by ants have been explored since the early 20th century. Myrmecochory was first scientifically systematized and studied by the botanist Sernander [5], who identified the elaiosome as the dispersal mediator attached to seeds and preferred by ants. Early research primarily involved expanding species lists and mapping distributions of myrmecochorous plants, alongside feeding experiments investigating the relationship between diaspore traits and ant preferences. Research progressed relatively slowly until the 1970s, when Berg [6] published the first study on myrmecochory in Australia, revealing that the seeds of approximately 1500 plant species in the region are dispersed by ants. In South Africa, Bond and Slingsby [7] conducted similar research, confirming that about 1000 plant species there are myrmecochorous.

Since the 1980s, research interest in myrmecochory has grown, leading to the emergence of studies that have focused on its evolutionary dynamics. In particular, as ecological data have accumulated on the principles of myrmecochory and the mechanisms underlying ant–plant mutualism, the three core hypotheses have become firmly established. Culver and Beattie [8] and Horvitz [9] challenged the distance-dispersal hypothesis, arguing that it provided little to no benefit—and potentially even negative effects—for myrmecochorous plants, although their claims were based on unverified assumptions. Later, Beattie and Kubitzki [10] and Beattie [11] investigated the diversity of myrmecochorous strategies and argued that the three main hypotheses—directed dispersal, distance dispersal, and predator avoidance—have all played significant roles in its evolution.

More recently, research has increasingly examined how elaiosomes influence ants under varying environmental conditions and how the benefits of myrmecochory for plants differ between ecosystems. In particular, studies have explored the impacts of invasive ants and plants on native myrmecochory, providing insights into how these interactions may be affected by anthropogenic disturbance. Nevertheless, the broader prospects of myrmecochorous interactions under anthropogenic environmental changes, as well as their impacts on biodiversity, have not been comprehensively explored. Moreover, there is a critical knowledge gap due to the scarcity of myrmecochory studies in East Asia, a region increasingly exposed to urbanization, climate change, and species invasions.

This review aims to (1) examine the ecological mechanisms of myrmecochory, (2) synthesize taxonomic information of myrmecochorous plants and ants, and (3) discuss future research directions in the context of urbanization and a climate change. Through this synthesis, we provide theoretical foundations, applied perspectives, and research strategies for understanding these ant–plant interactions.

## 2. Materials and Methods

We conducted a literature search on 4 December 2025, using Google Scholar and Scopus databases. The primary search employed the terms “myrmecochorous” and “myrmecochory,” initially yielding a total of 470 peer-reviewed articles in English. To align with the specific objectives of this review, we conducted two targeted searches: (1) impact of environmental disturbances on myrmecochory and (2) regional focus on temperate East Asia.

To explore the relationship between myrmecochory and contemporary stressors, we searched for articles using the following string: *TITLE-ABS-KEY ((myrmecochorous OR myrmecochory OR “Seed dispersal by ant” OR “ant-dispersal seed” OR “ant-mediated seed dispersal”) AND (“climate change” OR invasive OR urbanization OR “changing climate” OR warming OR disturbance OR alien))*. This search initially yielded 119 papers. We then applied a screening process that excluded studies where myrmecochory was only briefly mentioned; for example, several studies were excluded because they mentioned myrmecochory only for taxonomic descriptions of new species or first records without ecological analysis. Finally, 84 papers were selected for in-depth synthesis.

Given the relative scarcity of research in temperate East Asia compared to the tropics and the Southern Hemisphere, we conducted a regional search using the string: *TITLE-ABS-KEY ((myrmecochorous OR myrmecochory OR “Seed dispersal by ant” OR “ant-dispersal seed” OR “ant-mediated seed dispersal”) AND (Asia OR Korea OR Japan OR China OR Taiwan))*. The search returned 39 papers, which were narrowed down to 16 papers after applying the same screening criteria. Because this literature review specifically aimed to analyze ecological studies of myrmecochory within East Asian countries, we excluded publications that were not conducted in this region (e.g., Southeast Asia) or did not focus on the ecological aspects of myrmecochory. For instance, studies concerning walking stick insect eggs mimicking myrmecochorous seeds, genetic or successional analyses, and taxonomic reports of new *Viola* species were excluded from the selection. For each selected article, we extracted data on the study location, involved ant and plant species, research methodology, and key drivers of change (e.g., climate change, urbanization, and invasive species).

Furthermore, we identified and classified myrmecochorous ant genera by analyzing the Supplementary Material Supplementary Dataset from Luo et al. [12], which catalogs 338 plant-mutualistic ant genera characterized by the presence of domatia, extrafloral nectaries, or elaiosomes. We specifically isolated and quantified the subfamilies and genera associated with elaiosome collection. Finally, we reconstructed the phylogenetic tree of these myrmecochorous lineages based on the phylogenomic framework of Romiguier et al. [13].

We established a comprehensive checklist of myrmecochorous plant species in South Korea using a stepwise, systematic review approach. First, we compiled a baseline list of 37 species across 11 families previously documented as myrmecochorous in South Korean literature [14,15,16,17,18,19]. Second, expanding on this baseline, congeneric species within the verified genera were identified as potentially myrmecochorous species; we then verified the presence of diagnostic morphological traits (e.g., elaiosomes and/or caruncles) by examining high-resolution seed photographs from the National Species Database of Korea [20]. For instance, the myrmecochorous status of *Melampyrum koreanum* was confirmed by evaluating seed traits within *Melampyrum* spp., a genus where myrmecochory has been established for several relatives (e.g., *M. roseum* and *M. setaceum* var. *nakaianum).* Third, the search boundary was expanded to include myrmecochorous species documented in international studies that are also natively distributed in South Korea, followed by the same morphological trait verification. Finally, all native species of the genera *Viola* and *Corydalis* were comprehensively included, given the evolutionary conservatism and ubiquity of myrmecochory within these specific lineages as established by Beattie and Lyons [21] for *Viola* and *Fukuhara* [22] for *Corydalis*. Through this rigorous process, elaiosome-bearing relatives were systematically incorporated into the final dataset, even in the absence of explicit, published species-specific field reports.

## 3. Evolution of Myrmecochory

### 3.1. Hypotheses Describing Myrmecochorous Interactions

Myrmecochory is a seed dispersal strategy mediated by ants that is generally more efficient than dispersal that occurs through incidental handling or caching behavior. Myrmecochory is particularly common in the shrublands of the Southern Hemisphere, in South Africa and Australia, and in temperate forest ecosystems of the Northern Hemisphere [5,23]. In this example of selective mutualism, ants detach and consume only the elaiosome from the seed, with the remainder discarded either in the colony’s refuse pile or in the vicinity of the nest, where it subsequently germinates [24]. This interaction provides mutual benefits for the ants and plants: the ants obtain nutritional resources from the elaiosome [25,26], while the plants benefit from the dispersal of their seeds.

**Figure 1:**
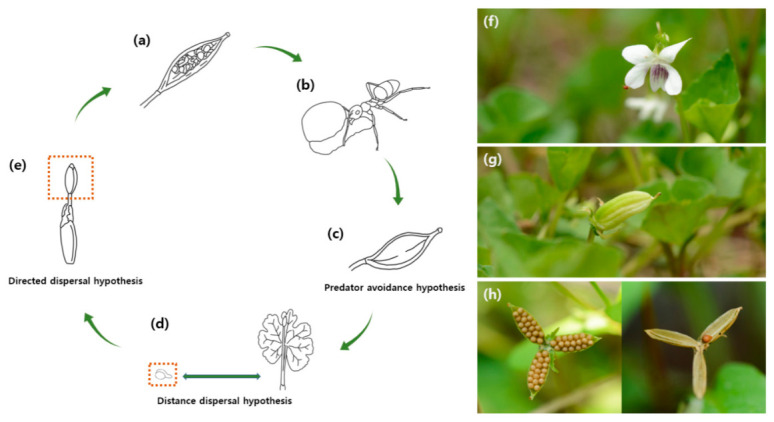
Schematic diagram of myrmecochory and the hypotheses underlying its emergence. The green arrows indicate the procedures of myrmecochorous dispersal of seeds, which are highlighted by the red-dashed box. (**a**) Seeds mature and fruit dehisce. (**b**) Ants disperse diaspores containing an elaiosome. (**c**) Predator avoidance hypothesis: Ant dispersal enables diaspores to avoid seed predators. (**d**) Distance dispersal hypothesis: Seeds are dispersed farther from the parent plant. (**e**) Directed dispersal hypothesis: Seeds germinate and grow in more favorable sites. (**f**–**h**) Photographs of *Viola verecunda* from flowering to seed dispersal via fruit maturation and dehiscence (Photo credit: Seongwon Yun).

While the benefits of myrmecochory for plants have been widely investigated [27], the advantages for ants remain relatively unexplored [28]. Because seed dispersal and germination are directly tied to plant reproduction, this interaction has a positive influence on plant adaptation and diversification. In particular, the selective advantages provided by ant-mediated diaspore dispersal have been explained using three major hypotheses: (1) directed dispersal, (2) distance dispersal, and (3) predator avoidance (Figure 1).

1. *Predator avoidance:* This hypothesis proposes that, when ants collect seeds before other animals, such as beetles, rodents, or birds, they effectively protect the seeds from predation [29,30,31]. Compared to endozoochory (dispersal via ingestion by vertebrates), myrmecochory often preserves the integrity of seeds, which may result in greater dispersal efficiency. According to this hypothesis, elaiosomes produce chemical compounds that attract ants, prompting them to retrieve the seeds rapidly. Although research on the chemical composition of these attractants remains limited, these studies have improved the understanding of the adaptive value and selective advantages of ant–plant mutualisms in myrmecochory [32,33]. Continued investigation into the properties of elaiosomes would clarify the proximate causes and the emergence of these myrmecochrous interactions.
2. *Distance dispersal*: By transporting seeds away from the parent plant, ants expand the spatial distribution of plant populations and reduce parent–offspring competition [34]. Although this hypothesis has been explored in temperate forests, it has been more frequently supported in heathlands and sclerophyll forests of the Southern Hemisphere, where myrmecochory is especially common. Studies have found a significant positive correlation between the body size of seed-dispersing ants and the distance over which seeds are transported [35,36]. Research has also reported a trend toward greater dispersal distances in regions with low mean annual precipitation, low primary productivity, and low ant colony densities. In addition, woody myrmecochorous plants in the Southern Hemisphere tend to be dispersed over greater distances than herbaceous myrmecochorous plants in temperate forests. These differences in dispersal patterns may be related to climate-driven variation in plant community composition, with seed-dispersing ants in non-temperate environments potentially maintaining lower colony densities or larger foraging ranges compared to those in temperate regions [37]. In tropical rainforests, myrmecochory is more often associated with herbaceous plants, but dispersal distances tend to be shorter [31], suggesting that the advantage of increased dispersal distance is less important in these environments.
3. *Directed dispersal*: This hypothesis proposes that ants transport plant seeds into or near their nests, thereby placing them in environments more favorable for germination [31]. The soils in and around ant nests undergo chemical and physical changes due to ant activity, creating conditions that are more conducive to seed germination and seedling growth [38]. Therefore, this hypothesis is considered to be particularly applicable to ant species that remain in a single location for extended periods and build prominent mounds. Empirical support for this mechanism is also stronger for sclerophyll forests and temperate open habitats [31]. In tropical rainforests, myrmecochory frequently occurs in the form of secondary dispersal, where the seeds of trees and shrubs are first dispersed by vertebrates and then by ants. However, because monitoring this dispersal process is labor-intensive and methodologically challenging and its outcomes are difficult to quantify, empirical studies remain relatively scarce [39].

In some ecosystems, fire avoidance and adaptation to nutrient-poor environments have also been proposed as additional benefits of myrmecochory. When ants transport seeds underground, they can be protected from fire damage [40]. Which of the three main hypotheses is more likely for a particular myrmecochorous relationship depends on the ecological characteristics of the seed-dispersing ant species, their habitat, and associated vegetation. It is also possible for multiple selective advantages to act simultaneously, making it difficult to isolate and independently evaluate each hypothesis [31].

### 3.2. Evolutionary Mechanisms of Myrmecochory

As with many other terrestrial organisms, the diversification of ants can be traced back to changes in Mesozoic flora, in particular the decline in the dominance of gymnosperms and the rise in angiosperms [41]. The emergence of new plant lineages increased ecosystem complexity and expanded the range of available resources. The high transpiration capacity of angiosperms also influenced the climate, leading to the expansion of tropical rainforests [42]. Due to these environmental changes, ants increasingly incorporated plant materials into their diet as they moved from the ground into arboreal habitats [43]. Compared to obligate carnivory, exploiting plant-derived resources requires less energy than capturing and subduing prey [44]. Plants also benefited from the ecosystem services provided by ants, leading to the development of mutualistic interactions and driving the diversification of both ants and plants. Consequently, both groups have evolved into some of the most diverse and abundant organisms on Earth, occupying a wide range of ecological niches.

It has been debated whether myrmecochory represents a truly mutualistic interaction between ants and plants. Given that seed dispersal and germination are directly linked to plant reproductive success, myrmecochorous plants may strongly depend on ants [45]. For ants, however, although elaiosomes are a relatively low-cost source of nutrients, they may not be a particularly attractive resource in ecosystems already rich in biodiversity and food availability. In addition, myrmecochory does not always function as a strict mutualistic relationship. It is uncommon for a plant species to rely exclusively on a single ant species for dispersal or for an ant species to transport seeds from only one plant species, suggesting that myrmecochory is a unilateral adaptation by plants to exploit the foraging behavior of ants [45]. Moreover, the selection of diaspores by ants is not solely driven by nutritional gain nor determined exclusively by traits such as diaspore size, ant body size, or the elaiosome-to-seed mass ratio. Rather, elaiosomes act as attractants that allow ants to detect and collect seeds before they are exposed to other seed predators, potentially enhancing colony fitness [46,47].

## 4. Taxonomy and Distribution of Myrmecochory

### 4.1. Myrmecochorous Plant Taxonomy and Elaiosome Morphology

Myrmecochory is distributed across all major biomes with the exception of Antarctica [48]. Lengyel et al. [49] estimated that this dispersal syndrome is employed by more than 11,000 plant species within 334 genera and 77 families, spanning a wide range of clades and orders worldwide (Table 1). A significant number of myrmecochorous species (>500 species per order) have been identified in the Piperales (Aristolochiaceae, Piperaceae), Alismatales (Araceae), Asparagales (e.g., Asphodelaceae, Laxmanniaceae), Ranunculales (e.g., Papaveraceae, Berberidaceae), Proteales (Proteaceae), Malpighiales (e.g., Violaceae, Picrodendraceae), Fabales (Fabaceae, Polygalaceae), Rosales (e.g., Rosaceae, Cannabaceae), Malvales (Malvaceae, Thymelaeaceae), Lamiales (e.g., Lamiaceae, Orobanchaceae), Asterales (e.g., Asteraceae, Goodeniaceae), and Ericales (e.g., Primulaceae, Ericaceae).

**Figure 2:**
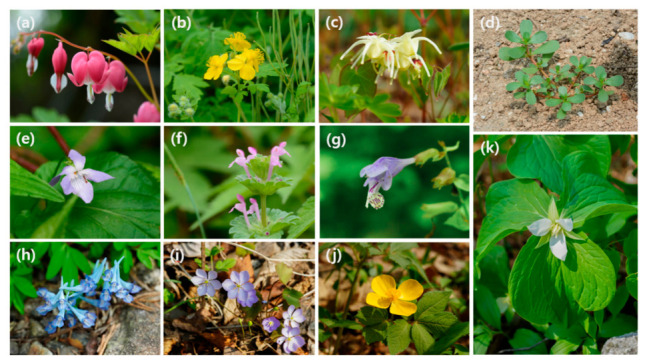
Representative myrmecochorous plants with elaiosomes in South Korea (**a**) *Lamprocapnos spectabilis*, (**b**) *Chelidonium majus* var. *asiaticum*, (**c**) *Epimedium koreanum*, (**d**) *Portulaca oleracea*, (**e**) *Viola acuminata*, (**f**) *Lamium amplexicaule*, (**g**) *Meehania urticifolia*, (**h**) *Corydalis ambigua*, (**i**) *Jeffersonia dubia*, (**j**) *Hylomecon vernalis*, and (**k**) *Trillium camschatcense*.

In South Korea, a representative region of temperate East Asia, we identified myrmecochorous plants spanning 14 orders, 21 families, 32 genera, and 130 species (Table 1, Figure 2). The Violaceae (50 spp.) are the most species-rich family, followed by the Papaveraceae (36 spp.). Notably, these species are almost exclusively perennial and/or spring ephemeral herbs. Interestingly, while a vast number of myrmecochorous plants have been reported in the Fabaceae (e.g., *Acacia*, *Pultenaea*) across the Southern Hemisphere, temperate East Asian Fabaceae functionally lack this dispersal syndrome.

However, this list of myrmecochorous plants remains tentative, with verification required. For example, most species in *Viola* and *Corydalis* are known to have elaiosomes; thus, all of the species in these genera have been included in the list, despite a few of these lacking evidence for the presence of an elaiosome. Conversely, many more unreported myrmecochorous species are possible for genera such as *Ajuga*, *Hemerocallis*, *Persicaria*, *Melica*, *Luzula*, *Iris*, and *Euphorbia* because species in these genera were only included in the myrmecochorous list when the presence of an elaiosome or caruncle structures was confirmed in past studies or through botanical photographs. As more morphological information on seeds becomes available, the number of myrmecochorous species may increase.

**Figure 3:**
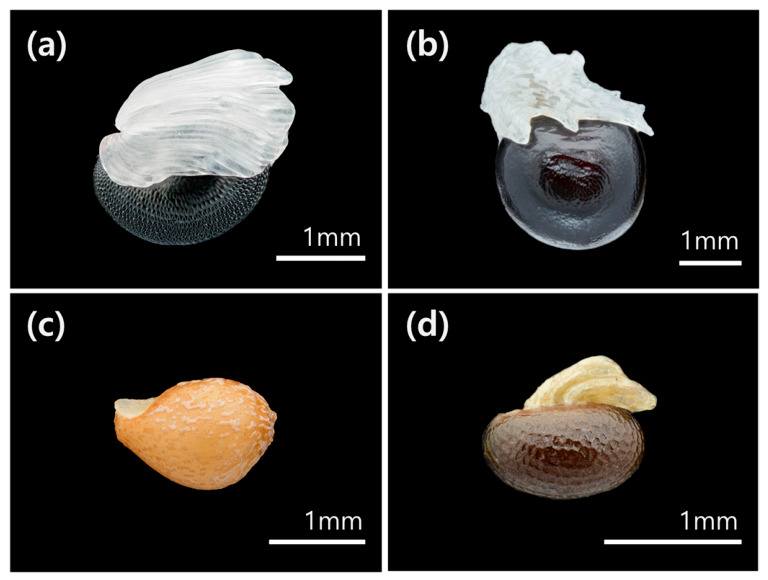
Morphological diversity of elaiosomes. (**a**) *Corydalis speciosa*, (**b**) *Lamprocapnos spectabilis*, (**c**) *Viola mandshurica*, and (**d**) *Chelidonium majus* var. *asiaticum* (Photo credit: Seongwon Yun).

The elaiosome in myrmecochorous plants attracts ants and serves as the dispersal mediator (Figure 3). Elaiosomes are fleshy appendages attached to seeds [50] that are composed mainly of lipids, proteins, and carbohydrates [51]. They primarily attract ants through diglycerides and triglycerides, which mimic insect prey [52,53]. Depending on the plant lineage, elaiosomes have developed from different seed tissues (e.g., chalazas, funiculi, hila, raphe–antiraphe) or fruit tissues (e.g., the exocarp, receptacles, flower tubes, perigonia, styles, or spicules) [54]. Despite this diversity of origins and developmental pathways, all elaiosomes share the same ultimate function of attracting ants and provide them with a nutritional reward, making myrmecochory a notable case of convergent evolution [4]. Mapping myrmecochorous plants within the angiosperm phylogeny reveals that they are more common in relatively young families (<70 million years), supporting the hypothesis that elaiosomes have consistently provided a selective advantage for plants since ants became abundant [49].

The mass proportion of elaiosomes in seeds and their chemical composition varies considerably between taxa [53], and even closely related species within the same genus can differ significantly in this respect [32]. Similarly, the shape and size of the elaiosome differ between species. For example, in *Corydalis speciosa*, the elaiosome covers a large surface area of the seed (Figure 3a), whereas, in *Lamprocapnos* spectabilis, it is a protruding structure covering only part of the seed (Figure 3b). In *Viola* species, elaiosomes are relatively small, possibly because their seeds undergo ballistic dispersal as their primary mechanism, followed by secondary dispersal via ants (Figure 3c). The size of the elaiosome can also influence ant preferences [33]; as the elaiosome-to-seed mass ratio increases, the probability that a seed will be taken by a seed-dispersing rather than seed-predating ant species rises [55]. Therefore, larger elaiosomes may have a higher likelihood of being selected by ants. However, in some cases, ants do not follow the expected elaiosome-to-seed ratio in selecting diaspores. This may occur when chemical attractants in the elaiosome override the ants’ ability to discriminate optimal ratios, disrupting their energetically optimal cost–benefit decision-making process [56].

### 4.2. Myrmecochorous Ant Taxonomy and Dependence on Elaiosomes

**Figure 4:**
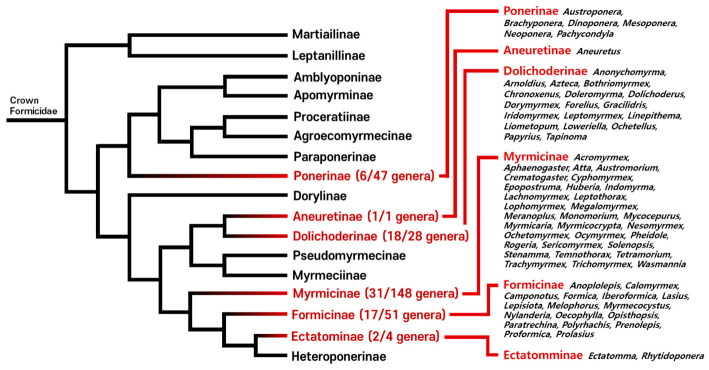
Phylogenetic distribution of myrmecochorous ant subfamilies (highlighted in red). The phylogenetic tree and raw data for myrmecochorous subfamilies were reconstructed from Romiguier et al. [13] and Luo et al. [12], respectively. Numbers in parentheses represent the ratio of reported myrmecochorous genera to the total number of genera within each subfamily.

By extracting the myrmecochorous ant genera present in the Supplementary Material Supplementary Dataset constructed by Luo et al. [12], we identified myrmecochorous ant species from 73 genera in six subfamilies (Myrmicinae, Formicinae, Dolichoderinae, Ponerinae, Ectatomminae, and Aneuretinae) in the present study (Figure 4). Myrmicinae (30 genera), Dolichoderinae (18 genera), and Formicinae (16 genera), which are some of the most species-rich ant lineages, have the highest number of myrmecochorous genera. According to Moreau [57] and Nelsen [58], these clades are characterized by the incorporation of plant-derived resources into their diets and represent major ant radiations that diversified alongside the expansion of angiosperms during the Mesozoic Era (Figure 4). On the other hand, fewer genera were found in Ponerinae (6 genera), which is a predominantly carnivorous subfamily in the Poneroid clade, due to their weak reliance on vegetative diets. In addition, three genera spanning Ectatomminae (2 genera) and Aneuretinae (1 genus) have been reported to be myrmecochorous (Figure 4).

When ants transport diaspores, the elaiosome may be fed to larvae or directly consumed by adult ants, providing essential nutrients such as vitamins, amino acids, and fatty acids [59]. Ants can thus obtain these nutrients at a lower cost by harvesting the elaiosomes of plant seeds rather than hunting other animals, and it has been suggested that the nutritional value of elaiosomes may become more significant in resource-limited environments or during early colony development, where alternative food is scarce. However, under conditions where other food sources are abundant, the preference for elaiosomes may decrease [60,61]; thus, the dependence of ants on these plants is generally considered to be lower than the dependence of the plants on ants [62]. In addition, myrmecochorous plants are estimated to represent only about 4.5% of all angiosperm species and roughly 17% of all angiosperm families [49], with seeds being available to ants only for a limited period. As a result, they may not exert a strong enough selective pressure to drive the evolution of specialized traits in ants.

While ants do not appear to have evolved morphological traits specifically adapted for myrmecochory, they can be classified into high- and low-quality dispersers based on their dietary preferences. High-quality dispersers rapidly transport seeds over greater distances, show a stronger preference for seeds, and favor diaspores with larger elaiosomes [36,63]. In contrast, low-quality dispersers either consume only the elaiosome without moving the seed or disperse it over short distances [64]. High-quality dispersers tend to be scavenger species that feed on dead insects while also collecting seeds, whereas low-quality dispersers are often granivorous species that primarily consume seeds [65]. Because the chemical composition of many elaiosomes resembles that of insect prey [52], it has been suggested that elaiosomes may play an important role in attracting high-quality dispersers [66].

The seed dispersal distances achieved via myrmecochory are estimated to range between 2 and 15 m, which is shorter than those typically achieved using anemochory or other forms of zoochory [67,68]. In particular, Gómez and Espadaler [37] experimentally measured myrmecochorous dispersal distances, reporting a mean of 2.24 ± 7.19 m. The longest recorded dispersal distance has been observed for *Iridomyrmex viridiaeneus* (Dolichoderinae) transporting *Acacia ligulata* seeds, reaching 180 m, with the second longest average dispersal distance recorded for the genus *Formica* [69].

**Figure 5:**
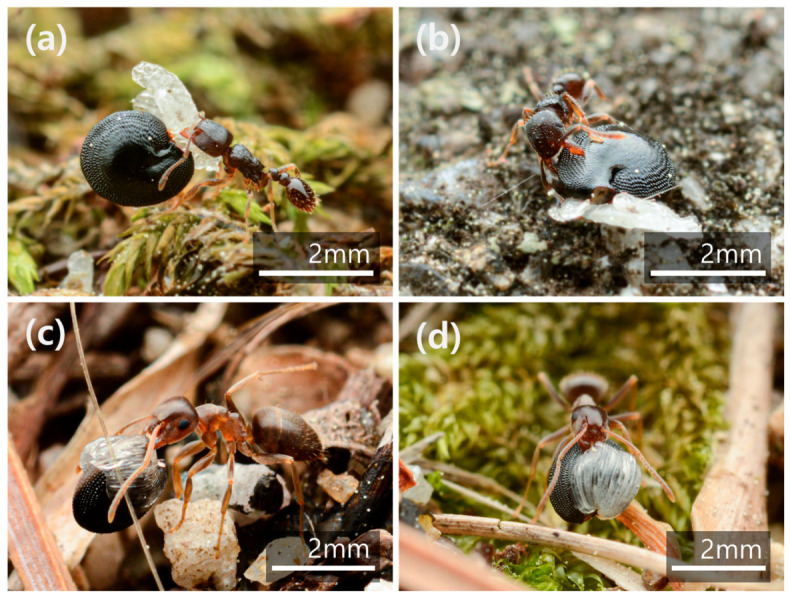
Images of ants of different sizes ((**a**,**b**): *Tetramorium tsushimae*; (**c**,**d**): *Lasius alienus*) transporting *Corydalis speciosa* diaspores. (**a**,**b**) The smaller *Tetramorium tsushimae* (length: 2.2–2.8 mm) experiencing difficulty in transporting a diaspore. (**c**,**d**) The larger *Lasius alienus* (length: 2.0–4.0 mm) exhibiting successful transportation (Photo credit: Seongwon Yun).

Seed selection by ants is generally influenced by the mass of the elaiosome. Numerous studies have demonstrated that diaspores with heavier elaiosomes and a higher elaiosome-to-seed mass ratio are more likely to be selected. Larger-bodied ant species tend to transport diaspores with larger elaiosomes [70,71,72], and the body size of disperser ants is positively correlated with the dispersal distance [34,36]. The size of the ant species can also limit the maximum diaspore size it can handle, resulting in species-specific preferences for certain plant taxa (Figure 5). Notably, smaller ants often cannot grip and move seeds once the elaiosome has been removed (Figure 5a,b), meaning that seeds of certain morphological types are left within the nest [24]. On the other hand, larger ants such as *Lasius alienus* can successfully transport diaspores further (Figure 5c,d).

In addition to body size, the ecological habits of ants can influence seed dispersal patterns [73]. For example, Gorb et al. [74] compared the dispersal patterns of diaspores by *Lasius fuliginosus* and *Formica polyctena*. In their study, *L. fuliginosus* deposited refuse near nest entrances along tree trunks, whereas *F. polyctena* deposited refuse along territorial boundaries. Seedling abundance and species diversity within the territory of *L. fuliginosus* wgoere slightly lower than in other sampled areas. These findings highlight that ant traits-including body size and nesting behavior-as well as seed morphology can influence the distribution patterns of myrmecochorous plants [75,76].

### 4.3. Distribution of Myrmecochory and Climatic Influences

Though myrmecochory is observed in most biomes worldwide, it is particularly common in dry heathlands and sclerophyllous vegetation in Australia (~1500 species) and the South African fynbos (~1000 species). According to Luo et al. [76], the species diversity and biomass of myrmecochorous plants generally increase toward lower latitudes and peak in mid-latitudes, while domatium-bearing plants are proportionally more abundant in tropical zones. Several studies have suggested that this distribution of myrmecochorous plants is linked to annual precipitation and primary productivity. In dry, low-precipitation environments, elaiosomes may be more energy-efficient for plants than other dispersal mechanisms because they require less potassium, which is scarce in the soils of these environments. In addition, in resource-limited environments, elaiosomes provide a readily collectible food source for ants, with lower energy costs for foraging and hunting. Disperser ant species also vary with elevation; thus, dispersal patterns may differ between lowland and highland areas [77]. Therefore, understanding dispersal systems at different elevations can also enhance the knowledge of plant distribution.

Myrmecochory also varies in terms of development and patterns depending on the environmental conditions. For example, sclerophyllous myrmecochores had longer seed dispersal distances (2.79 m) than mesophyllous myrmecochores (1.09 m) [37]. In addition, the mean seed dispersal distance for non-myrmecochorous plants in savannas is three times greater than that in tropical rainforests [78], likely due to climatic and geographical differences in their habitats. One possible explanation for these dispersal patterns is that, in climatic zones where vertebrate dispersers are scarce or where producing fleshy fruits is difficult, ants may serve as important dispersal agents. As such, myrmecochory may be favored by selection in open, dry, and unstable habitats rather than in closed, humid, and stable ones [49]. Given that myrmecochorous lineages exhibit more than twice the diversification rates of their sister groups on average, this hypothesis appears plausible [48], although further evidence is needed.

## 5. Approaches and Prospects for Myrmecochory Research

### 5.1. Approaches to Myrmecochory Research

Because the core process of myrmecochory involves ants transporting plant seeds and the subsequent germination of those seeds, most early studies focused on identifying the presence of elaiosomes and/or directly observing these interactions. Research has been conducted both in the field, targeting naturally growing plants, and in laboratory settings through controlled feeding experiments [79]. This section outlines some of the most important research methodologies employed for these purposes.

1. *Diaspore trait analysis*: This approach examines the characteristics of elaiosomes, such as their size and weight, followed by feeding experiments to determine which of these factors are preferred by ants [80]. Alternatively, chemical analysis of elaiosomes can be conducted to assess the proportion of lipids, proteins, volatiles, and other components and determine how this affects ant preferences. For example, Sasidharan and Venkatesan [50] evaluated the role of volatile signals from elaiosomes using Y-tube olfactometry and analyzed their chemical composition via gas chromatography.
2. *Soil analysis and germination experiments*: These experiments evaluate how the presence of an ant colony alters the physical and chemical properties of the soil and whether these differences affect seed germination rates. For example, Dostál et al. [38] collected soil samples from active ant mounds, adjacent soils, and abandoned ant mounds. They then analyzed the levels of Ca^2+^, K^+^, Mg^2+^, and available P, the C:N ratio, and pH and conducted germination trials to determine whether these differences significantly influenced plant establishment.
3. *Mesocosm and* in situ *experiments*: This method evaluates how ants influence the fate of seeds under semi-natural conditions. Fokuhl et al. [81] installed mesocosms designed to mimic natural environments, placing ant nests and mother plants at a set distance within fenced plots that excluded other animals. Experimental treatments tested the presence or absence of ants, with corresponding control plots. After a set period, researchers recorded the location and distance of germinated seedlings to assess how ant activity influenced seed dispersal and survival.

Myrmecochory has long been recognized as a mutualistic relationship, with both ants and plants assumed to benefit from the interaction. However, relatively few studies have examined the extent to which each partner gains from this relationship. In particular, research attention has been more strongly directed toward the advantages from the plant’s perspective, whereas studies explicitly determining the fitness benefits for ants remain limited. Because empirical findings on how elaiosomes influence ant colony productivity have been inconsistent [82], myrmecochory is generally perceived as a relationship that is more important for plants. For this reason, it is often referred to as a facultative mutualism rather than a strongly obligate one. From the plant’s perspective, there is also a lack of detailed understanding of what happens after seeds are dispersed, specifically, how they germinate and grow in the new location. This knowledge gap highlights the need for further research to fully evaluate the ecological and evolutionary implications of myrmecochory.

### 5.2. Myrmecochory Research in Temperate East Asia

A total of 16 myrmecochory studies conducted in temperate East Asia were analyzed (Table 2). Myrmecochory was primarily observed in forest understory herbs such as *Viola* spp., *Corydalis* spp., and *Erythronium* spp. The principal seed dispersers were ants belonging to the subfamilies Myrmicinae and Formicinae, specifically the genera *Myrmica*, *Lasius*, *Formica*, and *Aphaenogaster*.

Methodologically, most research relied on in situ observations involving manual tracking of dispersal distances [84,91]. Additionally, in situ experiments were frequently employed to manipulate access for ants, rodents, and other predators [92,97] and to adjust seed placement patterns [88,92]. These studies typically integrated diaspore trait analyses, including morphological measurements of seeds and elaiosomes as well as lipid composition studies. A limited number of studies utilized advanced techniques such as laboratory mesocosm experiments with artificial nests [90], fixed-point video recording [93], or molecular marker analysis (e.g., ISSR) to evaluate spatial genetic structures [94].

This overview suggests that myrmecochory research in East Asia remains underdeveloped. Despite several pioneering studies in Japan and China, various myrmecochorous plant and ant species across diverse habitats—including alpine areas, grasslands, and urban environments—remain largely uninvestigated. Consequently, there are significant opportunities for future research to expand our understanding of myrmecochory in this region.

Beyond basic documentation, comprehensive research is essential to elucidate the role of myrmecochory in maintaining ecological integrity and promoting natural regeneration, highlighting its vital importance for biodiversity conservation and ecosystem restoration. In South Korea, we identified 4 endangered, 18 rare, and 12 endemic myrmecochorous species (Table A2), most of which belong to the genera *Viola* and *Corydalis*. The role and ecological significance of myrmecochory in the distribution and regeneration of these species remain unclear. From a biodiversity conservation perspective, future research should first prioritize identifying undiscovered endangered myrmecochores and establishing their specific ant partners. Concurrently, testable experimental designs and long-term field monitoring—including controlled laboratory feeding experiments and morphological/chemical analyses of elaiosomes—are required to rigorously evaluate the functional dependencies, threats, and evolutionary benefits within these mutualisms.

### 5.3. Myrmecochory Research Associated with Biodiversity, Urbanization, and Climate Change

**Figure 6:**
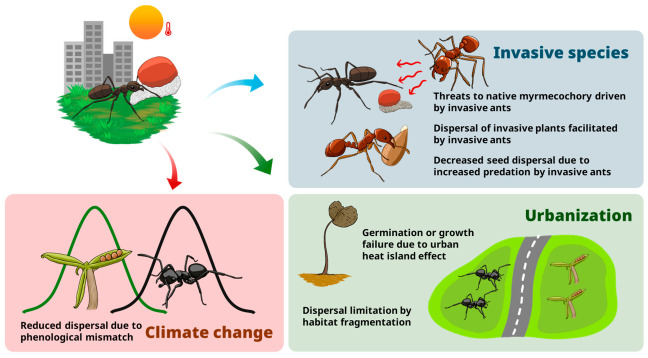
Hypothetical scenarios of myrmecochory changes under urbanization and climate change.

Our synthesis suggests threats of myrmecochory by species invasion, urbanization, and climate change (Figure 6). For myrmecochorous plants that rely entirely on ants for seed dispersal, the introduction of non-native ant species can dramatically alter their distribution patterns. For example, if an introduced ant species does not engage in seed dispersal, it may displace native disperser ants, disrupting the seed dispersal process for these plants [98]. As a result, plant populations may gradually decline and their distribution range may shrink; the larger the area occupied by invasive ants, the more severe the problem becomes. For example, invasive ants such as *Linepithema humile* and *Myrmica rubra* have also become problematic in Australia and North America because they actively disperse plant seeds of invasive plants over those of native species, and they have been found to disperse seeds over longer distances than native ants [99,100,101]. This behavior ultimately enhances the survival and spread of invasive plant species in these regions [102]. Conversely, there are cases where invasive ant species disperse seeds, but primarily those of alien plant species. This is referred to as secondary invasion, and it has been widely studied due to its potentially significant impacts on native ecosystems.

In the face of climate change and urbanization, changes in ant distribution and consequently myrmecochory are likely to vary between climatic zones. Ants in temperate regions generally have a higher tolerance to low temperatures by maintaining metabolic activity well above standard metabolic rates under cold stress and producing cryoprotectants to prevent cellular freezing. In contrast, although tropical ants possess a higher baseline tolerance to elevated temperatures, they may lack resilience to extreme heat, increasing their vulnerability under climate change scenarios [103]. In tropical regions, the combined effects of urban heat islands and elevated temperatures could push ants beyond their critical thermal maximum (*CT_max_*), affecting their survival and distribution. Conversely, warming in temperate regions may allow some species to expand their ranges [104].

If climate change leads to phenological mismatches, disrupting the synchronization between plant seed-setting periods and ant activity cycles, the seed dispersal behavior of ants may decline [105]. This risk is compounded when the climatic responsiveness of plants and ants differs. For example, a transplant experiment in Georgia, USA, suggested asynchrony between warm-adapted ants and early-blooming plants, which limited their myrmecochorous seed dispersal distance under shifting climatic conditions [106]. Such impacts are particularly severe for species with localized distributions or those inhabiting alpine regions. A notable example is the mutualism between the cold-adapted ant *Formica gagatoides* and the alpine threatened plant *Dicentra peregrina*, documented above the tree line in the Japanese Alps [84]. Given that these high-altitude groups often include endangered or protected species, prioritizing the monitoring of climate-driven shifts in myrmecochory is crucial for the long-term conservation of these rare taxa.

Urbanization processes intensify the fragmentation and loss of natural habitats worldwide [107], leading to the local extinction of many native species [108,109,110]. Development-driven disturbances also alter various ecosystem components, including the vegetation, soil, and insect community composition. In these altered environments, ants can play an important role as seed dispersers. Ants are one of the most dominant taxonomic groups in many terrestrial ecosystems and are among the most common arthropods found in both forests and urban areas [104,111]. Their small size and high species diversity even within small areas [111,112] enable them to maintain their function as seed dispersers in disturbed and fragmented ecosystems.

An increase in the number and diversity of effective seed-dispersing ant species could potentially enhance myrmecochory in disturbed habitats. However, these changes may also facilitate the spread and establishment of invasive species, ultimately leading to declines in native myrmecochorous interactions. Because the distribution of myrmecochorous plants depends on disperser ants, it is important in anthropogenically disturbed environments to understand which traits of seed dispersers are favored. The ecological services provided by ants depend heavily on the species composition of the ant community; thus, myrmecochory can be either promoted [113] or hindered [114,115] by urbanization.

Urbanization often reduces vegetation cover, decreasing the forest canopy and increasing the amount of sunlight reaching the ground. In addition, anthropogenic heat from vehicles and factories, along with impervious surfaces such as concrete and asphalt, contributes to the urban heat island effect. This creates high-temperature, low-humidity environments that may induce desiccation stress [116,117], favoring ant species that are physiologically adapted to higher temperatures and lower moisture conditions. Crucially, global climate change is expected to intensify these urbanization-driven threats, such as localized urban heat island effects, through interactive and synergistic effects.

Ecologically, these simplified communities tend to be dominated by omnivorous ants with broad foraging ranges that prefer open habitats. Consequently, the composition of ant species in urban areas differs from that in typical forests, and colony densities may also vary, with urbanization acting as a filter for the ant species composition [116]. For example, in areas with high vegetation cover, omnivorous predators, including ants, have a negative correlation with the proportion of green space [118]. In addition, Menke et al. [116] analyzed ant community composition changes along an urbanization gradient in the southeastern United States, finding that (1) urban ants were more tolerant of higher temperatures and lower moisture conditions, (2) drought-tolerant species dominated highly urbanized areas, and (3) the invasive species *Linepithema humile* was most abundant in moderately urbanized areas.

These anthropogenic changes in ant community composition can negatively affect myrmecochory [119]. For example, urban development leads to habitat fragmentation with edge effects, resulting in dominant or invasive species becoming more prevalent [120,121,122]. In these ecosystems, invasive ants may disrupt the close interactions between native ants and plants, negatively affecting myrmecochorous plants [123,124] by either dispersing more seeds of invasive plants or displacing native seed-dispersing ants, thus reducing dispersal [101]. However, in some cases, the establishment of invasive species does not negatively affect dispersal [125].

Conversely, there are also cases where urbanization can have a positive impact on myrmecochory. Thompson and McLachlan [113] investigated *Viola pubescens* in urban forests in Manitoba, Canada, and found that, despite lower ant diversity and simplified communities, dispersal of *V. pubescens* seeds increased. This was ascribed to changes in the community composition, where the ants remaining in urban forests were competitively dominant species that acted as more effective seed dispersers. Similarly, *Formica fusca* can persist in urban forests and, in the absence of other dominant species, can experience competitive release [126] and become more active as effective foragers for seed dispersal [127]. Another example is a study in Australia that examined the relationship between roadside disturbance and myrmecochory, finding that the dispersal distance increased from 69 m to 120 m after road construction [128].

While these findings provide insights into how myrmecochory changes following anthropogenic disturbance, differences in the climate, flora, and ant fauna between regions mean that more research is needed in East Asia. Considering the unexpectedly high diversity of myrmecochorous plants identified in South Korea, the lack of ecological studies in East Asia represents a substantial knowledge gap. Given the increasing frequency of development-related disturbances, myrmecochory may contribute to vegetation recovery in urban and other anthropogenically disturbed environments. In rapidly urbanizing East Asian countries, it may function as an important ecological interaction. Although research on the ecosystem functions provided by ants is extensive [129], studies on their roles in urban environments have been limited [104]. Moreover, most research on urban ant distribution and ecosystem service provision is concentrated in the United States and other Western countries, with a lack of studies from East Asia, Southeast Asia, tropical regions, and Africa [104]. Such regional imbalances in research inevitably lead to a knowledge bias regarding fundamental aspects of myrmecochory, including dispersal distances, specific interacting agents (flora and fauna), and their unique ecological functions. We believe this review will serve as a timely catalyst to stimulate myrmecochory research in these less-explored regions.

## 6. Conclusions

This review examined the mutual benefits that ants and plants obtain from myrmecochory, how this interaction operates within ecosystems, and its effects on plant distribution and biodiversity conservation. The three major hypotheses—directed dispersal, distance dispersal, and predator avoidance—explain the selective advantages gained by plants, and together they function as complementary mechanisms. In addition, we compiled taxonomic information on myrmecochorous plants and ants, updating the checklist of Korean myrmecochorous plants to 130 species and reclassifying them into endemic, rare, and endangered species.

Myrmecochory is currently facing the combined pressures of urbanization and climate change. Urbanization alters ant communities through habitat fragmentation, loss of green space, changes in soil structure, and the urban heat island effect, thus potentially disrupting existing myrmecochorous relationships. At the same time, dominant ants in disturbed habitats may serve as effective seed dispersers. This implies that even in simplified communities, the settlement of alternative seed dispersers adapted to the environment allows for plasticity, ensuring that dispersal is not interrupted and can adapt in a different direction. Climate change also modifies ant activity and distribution; in temperate regions, warming may increase dispersal potential, whereas in tropical regions, interactions are at greater risk of weakening due to ants’ vulnerability to extreme heat. Because urbanization and climate change operate simultaneously, myrmecochory is likely to show signs of both vulnerability and resilience depending on the specific ecological circumstances.

Within this context, myrmecochory holds particular importance for conservation ecology, yet it has historically received less attention than other plant–insect interactions. Given that many endemic, rare, and endangered plants depend on ant-mediated dispersal, conservation strategies must address not only seed production and germination but also the dispersal process and the stability of the ant communities associated with it. This highlights the need to conserve not only individual plant species but also the plant–ant interactions upon which they rely.

To achieve this, we propose the following strategic framework for myrmecochory conservation. First, expanding and refining taxonomic checklists remains a fundamental priority, particularly to overcome existing regional knowledge biases; this requires an interdisciplinary approach that bridges the domains of botany and entomology to capture a holistic understanding of ant–plant associations. Second, future research must shift its focus toward functional dynamics, environmental requirements, preferences, and network dependencies through integrated field monitoring and laboratory experiments. In particular, long-term observations on post-dispersal seed germination and seedling establishment are essential to quantitatively evaluate the degree of myrmecochory dependence among plant species. Third, habitat suitability for myrmecochory-dependent species will be predicted under future climate change and conservation scenarios by incorporating biotic interaction principles, rather than relying solely on abiotic variables. Ultimately, these integrated approaches will enable conservationists to devise far more robust and proactive management strategies for vulnerable plant species in rapidly changing ecosystems.

## Figures and Tables

**Table 1:** Global phylogenetic distribution of myrmecochorous plant families and their regional representation in South Korea.

Clade (APG IV)	Order	Family	Global No. of Species [49]	No. of Species in South Korea ^†^
Magnoliids	Piperales	Aristolochiaceae, Piperaceae	1686	1
Monocots	Alismatales	Araceae	1524	-
	Pandanales	Stemonaceae	27	-
	Liliales	Liliaceae, Melanthiaceae, Colchicaceae, Smilacaceae	389	4(3)
	Asparagales	Amaryllidaceae, Asphodelaceae, Iridaceae, Hypoxidaceae, Tecophilaeaceae, Laxmanniaceae	779	3
	Poales	Poaceae, Cyperaceae, Juncaceae, Restionaceae, Bromeliaceae, Dasypogonaceae	168	3
	Zingiberales	Zingiberaceae, Marantaceae, Costaceae	125	-
Eudicots (Basal)	Ranunculales	Papaveraceae, Berberidaceae, Ranunculaceae	1034	38(11)
	Proteales	Proteaceae	568	-
	Buxales	Buxaceae	57	-
	Dilleniales	Dilleniaceae	124	
Superrosids (Fabids)	Malpighiales	Violaceae, Euphorbiaceae, Phyllanthaceae, Picrodendraceae, Clusiaceae, Achariaceae	3381	58(13)
	Fabales	Fabaceae, Polygalaceae	3368	4(1)
	Rosales	Rosaceae, Cannabaceae, Urticaceae, Rhamnaceae	655+	1
	Oxalidales	Elaeocarpaceae	150	-
	Celastrales	Celastraceae	3	-
Superrosids (Malvids)	Cucurbitales	Caricaceae	23	
	Malvales	Malvaceae, Thymelaeaceae	503	-
	Brassicales	Gyrostemonaceae, Resedaceae, Cleomaceae	308	-
	Sapindales	Sapindaceae, Rutaceae, Nitrariaceae	192	-
	Myrtales	Myrtaceae, Penaeaceae, Melastomataceae	30	-
Superasterids	Caryophyllales	Caryophyllaceae, Polygonaceae, Portulacaceae, Amaranthaceae, Aizoaceae, Cactaceae, Limeaceae	460+	2(1)
	Santalales	Santalaceae, Loranthaceae, Balanophoraceae	179	1
	Ericales	Primulaceae, Ericaceae, Balsaminaceae, Marcgraviaceae	1396	1
Asterids (Lamiids)	Lamiales	Lamiaceae, Orobanchaceae, Scrophulariaceae, Gesneriaceae, Plantaginaceae, Oleaceae	1148	12
	Gentianales	Apocynaceae, Rubiaceae	303	-
	Solanales	Solanaceae	31	-
	Boraginales	Boraginaceae	50	1
Asterids (Campanulids)	Aquifoliales	Aquifoliaceae	1	-
	Bruniales	Bruniaceae	74	-
	Asterales	Asteraceae, Goodeniaceae, Campanulaceae, Menyanthaceae	1367	1
	Apiales	Apiaceae	1	-
	Dipsacales	Caprifoliaceae	143	-

^†^ This study (see details in Table A1). Numbers in parentheses indicate the sum of endangered, rare, or endemic species designated in South Korea.

**Table 2:** Overview of myrmecochory studies in East Asia.

Source	Study Site	Target Plant Species	Target Ant Disperser	Methodology
Higashi et al. [83]	Hokkaido, Japan	*Trillium tschonoskii*	*Myrmica ruginodis*, *Aphaenogaster japonica*, *Lasius niger*, *Camponotus obscuripes*, *Pheidole fervida*, *Leptothorax* sp.	In situ observation, soil analysis
Komatsu et al. [84]	Mt. Norikura, Japan	*Dicentra peregrina*	*Formica gagatoides*	In situ observation, diaspore trait analysis
Matsumoto et al. [85]	Okayama, Japan	*Pinellia tripartita*, *Viola mandshurica*, *Oxalis dillenii*	*Formica japonica*, *Tetramorium tsushimae*, *Pheidole nodus*	In situ observation
Nakanishi [86]	SW Japan	*Corydalis* (7 spp.)	Not available	Diaspore trait analysis
Ohkawara and Higashi [87]	Hokkaido, Japan	*Viola selkirkii*, *Viola verecunda*	*Myrmica kotokui*, *Lasius niger*	In situ experiment, diaspore trait analysis
Ohkawara et al. [29]	Hokkaido, Japan	*Erythronium japonicum*	*Myrmica kotokui*	In situ experiment
Ohkawara et al. [88]	Hokkaido, Japan	*Corydalis ambigua*	*Myrmica kotokui*, *Lasius japonicus*	In situ observation
Ohnishi et al. [89]	Kobe/Saga, Japan	*Chamaesyce maculata*	*Tetramorium tsushimae*, *Pheidole* *nodus*	In situ observation
Ohnishi et al. [90]	Saga, Japan	*Chamaesyce maculata*	*Tetramorium tsushimae*, *Pheidole* *nodus*	Lab experiment
Tanaka and Tokuda [91]	Saga, Japan	*Carex lanceolata*, *Carex tristachya*	*Formica japonica*, *Pheidole nodus*	In situ observation
Wang et al. [92]	Jilin, China	*Erythronium japonicum*	*Pristomyrmex punctatus*, *Lasius fuliginosus*, *Formica japonica*, *Lasius alienus*, *Myrmica lobicornis*, *Formica altayensis*, *Leptothorax galeatus*, *Plagiolepis rothneyi*, *Monomorium pharaonis*	In situ experiment, diaspore trait analysis
Watanabe-Toma and Ohi-Toma [93]	Shikoku, Japan	*Aristolochia kaempferi*	*Formica* sp.	In situ observation, camera recording
Zhou et al. [94]	Yunnan, China	*Globba lancangensis*	*Odontoponera transversa*, *Pachycondyla luteipes*, *Pheidole watsoni*, *Pheidole capellini*, etc.	In situ experiment, molecular marker analysis
Zhu et al. [95]	Shaanxi, China	*Luzula plumosa*, *Corydalis pseudoincisa*, *Epimedium pubescens*, *Helleborus thibetanus*	*Myrmica ruginodis*	In situ experiment
Zhu and Wang [96]	Shaanxi, China	*Corydalis giraldii*	*Lasius alienus*, *Formica fusca*, *Aphaenogaster smythiesii*, *Formica polyctena*, *Myrmica* sp.	In situ experiment, diaspore trait analysis
Zhu and Wang [97]	Shaanxi, China	*Epimedium pubescens*, *Helleborus thibetanus*	*Myrmica ruginodis*, *Paratrechina* sp1, *Paratrechina* sp2, *Tetramorium* sp., *Temnothorax* sp.	In situ experiment, diaspore trait analysis

## Data Availability

The original contributions presented in this study are included in the article/Supplementary Material. Further inquiries can be directed to the corresponding author.

## Supplementary Materials

The following supporting information can be downloaded at: https://www.mdpi.com/article/10.3390/insects17070677/s1, Supplementary dataset for the checklist of myrmecochorous plants in South Korea.

## Appendix A

**Table A1:** List of myrmecochorous plants in South Korea.

Order	Family	Genera	Representative Species
Asparagales	Asphodelaceae	*Hemerocallis*	*H. fulva* f. *kwanso*, *H. dumortieri*
	Iridaceae	*Iris*	*I. rossii*
Asterales	Asteraceae	*Silybum*	*S. marianum*
Boraginales	Boraginaceae	*Bothriospermum*	*B. tenellum*
Caryophyllales	Caryophyllaceae	*Moehringia*	*M. lateriflora*
	Portulacaceae	*Portulaca*	*P. oleracea*
Ericales	Primulaceae	*Primula*	*P. jesoana*
Fabales	Polygalaceae	*Polygala*	*P. japonica*, *P. sibirica*, *P. tatarinowii*, *P. tenuifolia*
Lamiales	Lamiaceae	*Ajuga*	*A. decumbens*
		*Lamium*	*L. amplexicaule*, *L. album* var. *barbatum*
		*Meehania*	*M. urticifolia*
		*Prunella*	*P. vulgaris* var. *lilacina*
	Orobanchaceae	*Melampyrum*	*M. roseum*, *M. roseum* var. *ovalifolium*, *M. koreanum*, *M. roseum* var. *japonicum*, *M. setaceum*, *M. setaceum* var. *nakaianum*
		*Pedicularis*	*P. resupinata*
Liliales	Liliaceae	*Erythronium*	*E. japonicum*
		*Tricyrtis*	*T. macropoda*
	Melanthiaceae	*Trillium*	*T. camschatcense*, *T. tschonoskii*
Malpighiales	Euphorbiaceae	*Acalypha*	*A. australis*
		*Euphorbia*	*E. ebracteolata*, *E. esula*, *E. helioscopia*, *E. jolkinii*, *E. pekinensis*, *E. sieboldiana*, *E. maculata*
	Violaceae	*Viola*	*V. acuminata*, *V. albida*, *V. albida* var. *chaerophylloides*, *V. albida* var. *takahashii*, *V. betonicifolia* var. *albescens*, *V. biflora*, *V. blandaeformis*, *V. boissieuana*, *V. collina*, *V. diamantiaca*, *V. dissecta*, *V. grypoceras*, *V. grypoceras* var. *exilis*, *V. hirtipes*, *V. hondoensis*, *V. ibukiana*, *V. inconspicua*, *V. japonica*, *V. kapsanensis*, *V. keiskei*, *V. kusanoana*, *V. lactiflora*, *V. mandshurica*, *V. mirabilis*, *V. mongolica*, *V. obtusa*, *V. orientalis*, *V. ovato-oblonga*, *Viola ovato-oblonga* f. *pubescens*, *V. patrinii*, *V. phalacrocarpa*, *V. phalacrocarpa* f. *glaberrima*, *V. philippica*, *V. raddeana*, *V. rossii*, *V. sacchalinensis*, *V. selkirkii*, *V. seoulensis*, *V. takesimana*, *V. tenuicornis*, *V. thibaudieri*, *V. tokubuchiana* var. *takedana*, *V. ulleungdoensis*, *V. variegata*, *V. verecunda*, *V. verecunda* var. *semilunaris*, *V. violacea*, *V. websteri*, *V. woosanensis*, *V. yazawana*
Piperales	Aristolochiaceae	*Asarum*	*A. sieboldii*
Poales	Juncaceae	*Luzula*	*L. capitata*, *L. multiflora*
	Poaceae	*Melica*	*M. nutans*
Ranunculales	Berberidaceae	*Epimedium*	*E. koreanum*
		*Jeffersonia*	*J. dubia*
	Papaveraceae	*Chelidonium*	*C. majus* var. *asiaticum*
		*Corydalis*	*C. alata*, *C. albipetala*, *C. ambigua*, *C. bungeana*, *C. buschii*, *C. caudata*, *C. cornupetala*, *C. decumbens*, *C. filistipes*, *C. gigantea*, *C. grandicalyx*, *C. hallaisanensis*, *C. heterocarpa*, *C. hirtipes*, *C. humilis*, *C. incisa*, *C. maculata*, *C. misandra*, *C. namdoensis*, *C. ochotensis*, *C. ohii*, *C. pallida*, *C. pauciovulata*, *C. platycarpa*, *C. raddeana*, *C. remota*, *C. speciosa*, *C. ternata*, *C. turtschaninovii*, *C. turtschaninovii* f. *pectinata*, *C. turtschaninovii* var. *linearis*, *C. wandoensis*
		*Lamprocapnos*	*L. spectabilis*
		*Fumaria*	*F. officinalis*
		*Hylomecon*	*H. vernalis*
Rosales	Cannabaceae	*Cumulus*	*H. scandens*
Santalales	Santalaceae	*Thesium*	*T. chinense*

**Table A2:** List of endangered, rare, and endemic myrmecochorous plant species in South Korea. Endangered and endemic species follow the guidelines of the National Institute of Biological Resources, while rare species follow those of the Korea National Arboretum.

Class	Species
Endangered	*Viola raddeana*, *Viola mirabilis*, *Viola websteri*, *Viola biflora*
Rare	*Moehringia lateriflora*, *Polygala tenuifolia*, *Tricyrtis macropoda*, *Trillium camschatcense*, *Trillium tschonoskii*, *Viola albida*, *Viola diamantiaca*, *Viola raddeana*, *Viola mirabilis*, *Viola websteri*, *Viola biflora*, *Viola boissieuana*, *Viola ibukiana*, *Viola kapsanensis*, *Viola thibaudieri*, *Epimedium koreanum*, *Jeffersonia dubia*, *Corydalis filistipes*
Endemic	*Viola kapsanensis*, *Viola seoulensis*, *Viola takesimana*, *Viola woosanensis*, *Corydalis filistipes*, *Corydalis cornupetala*, *Corydalis grandicalyx*, *Corydalis maculata*, *Corydalis alata*, *Corydalis albipetala*, *Corydalis hallaisanensis*, *Corydalis misandra*
